# Keishibukuryogan Reduces Renal Injury in the Early Stage of Renal Failure in the Remnant Kidney Model

**DOI:** 10.1093/ecam/nep089

**Published:** 2011-05-03

**Authors:** Takako Nakagawa, Izumi Tashiro, Makoto Fujimoto, Michiko Jo, Shinya Sakai, Hiroshi Oka, Hirozo Goto, Yutaka Shimada, Naotoshi Shibahara

**Affiliations:** ^1^Department of Kampo Diagnostics, Institute of Natural Medicine, University of Toyama, 2630 Sugitani, Toyama 930-0194, Japan; ^2^Department of Japanese Oriental Medicine, Faculty of Medicine, University of Toyama, Toyama, Japan; ^3^21st Century COE Program, University of Toyama, Toyama, Japan

## Abstract

The effects of keishibukuryogan on the early stage of progressive renal failure were examined in rats subjected to 5/6 nephrectomy. Keishibukuryogan, one of the traditional herbal formulations, was given orally at a dose of 1% (w/w) and 3% (w/w) in chow. Administration of keishibukuryogan was started at 1 week after 5/6 nephrectomy and was continued for 4 weeks. At the end of the experiment, Azan staining did not reveal any severe histological changes in the kidneys of the nephrectomized rats. On the other hand, significant increases in mRNA expressions of transforming growth factor-**β**_1_ and fibronectin related to tissue fibrosis, as examined by Reverse Transcriptase-Polymerase Chain Reaction, were observed in nephrectomized rats, and they were significantly suppressed by 3% keishibukuryogan treatment. Against gene expressions related to macrophage infiltration, 3% keishibukuryogan treatment significantly suppressed osteopontin mRNA levels, and monocyte chemoattractant protein-1 and vascular cell adhesion molecule-1 mRNA levels showed a tendency to decrease, but without statistical significance. It was also observed that 3% keishibukuryogan attenuated serum urea nitrogen and urinary protein excretion levels. From these results, it was suggested that keishibukuryogan exerts beneficial effects that result in slowing the progression of chronic renal failure.

## 1. Introduction

Chronic renal failure (CRF) is one of the serious health problems that are linked to increasing incidence and prevalence as well as a reduction in the quality of life and a rise in the cost of its care. Current therapy with agents that block the renin-angiotensin system and exert protein restriction are commonly employed management strategies of renal failure [1]. However, the number of patients with renal failure, especially patients undergoing hemodialysis and facing end-stage renal failure, is increasing worldwide. Therefore, searches for new effective therapeutic approaches are needed for CRF, including the use of alternative medicines such as traditional herbal medicine.

Traditional Chinese/Japanese medicine has a long history and has contributed to the prevention and treatment of various diseases. Keishibukuryogan was first mentioned in “Kinki-Yohryaku” (Jin-Gui-Yao-Lue), a classic text of Chinese medical science written in the 3rd century AD. Keishibukuryogan has been widely administered to patients with blood stagnation for improving blood circulation, and is now one of the most frequently used prescriptions in Japan. It was reported that keishibukuryogan improves conjunctional microcirculation in patients with cerebro-spinal vascular diseases [2], suggesting the contribution of its improving effects on hemorheological parameters such as blood viscosity, RBC aggregability and RBC deformability [3–5]. Moreover, Matsumoto et al. [6] have explored a proteomic approach for the diagnosis of blood stasis in rheumatoid arthritis patients treated with keishibukuryogan. In animal experiments, keishibukuryogan was reported to prevent the progression of atherosclerosis, and to preserve vascular endothelial function in cholesterol-fed rabbits and in diabetic rats [7, 8]. Recently, keishibukuryogan was revealed to suppress fibronectin deposition associated with transforming growth factor-*β*_1_ (TGF-*β*_1_) in the kidney of diabetic rats [9]. In this connection, to unearth additional possible effects of keishibukuryogan on CRF, we conducted the present study to examine its effects on the early stage of renal failure induced by 5/6 nephrectomy.

## 2. Methods

### 2.1. Test Drug

**Figure 1:**
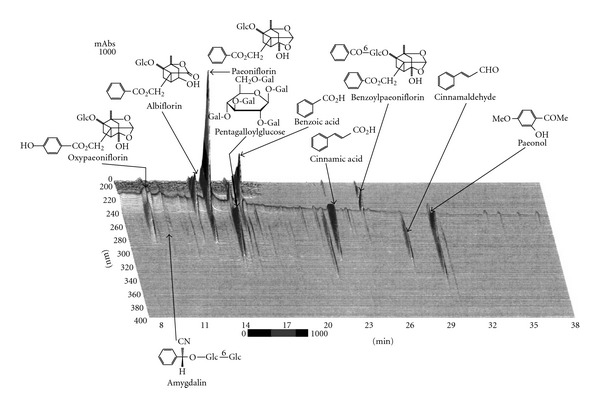
Three-dimensional HPLC profile of MeOH extract of keishibukuryogan. Glc, *β*-d-glucopyranosyl; Gal, galloyl.

Powdered keishibukuryogan was purchased from Uchida Wakanyaku (Tokyo, Japan). It is composed of equal parts, by weight, of the following five crude drugs: Cinnamomi Cortex, bark of *Cinnamomum cassia* Blume (Lauraceae); Poria, sclerotium of *Poria cocos* Wolf (Polyporaceae); Moutan Cortex, bark of *Paeonia suffruticosa* Andrews (Paeoniaceae); Persicae Semen, kernel of *Prunus persica* Batsch (Rosaceae) and Paeoniae Radix, root of *Paeonia lactiflora* Palas (Paeoniaceae). These crude drugs were mixed and shattered by grinding machine.  Figure 1 shows the 3D HPLC chart of keishibukuryogan. The HPLC conditions were as follows: keishibukuryogan extract (2.5 g) was obtained with 20 ml of methanol under ultrasonication for 30 min. The solution was passed though a membrane filter (0.45 *μ*m) and then subjected to HPLC analysis. HPLC equipment was controlled with an SLC-10A (Shimadzu, Tokyo, Japan) using a TSK-GEL ODS-80TS column (4.6 *ψ* × 250 mm), eluting with solvents (A) 0.05 M AcONH_4_ (pH 3.6) and (B) CH_3_CN. A linear gradient of 100% A and 0% B, changing over 60 min to 0% A and 100% B, was used. The flow rate was controlled with an LC-10AD pump at 1.0 ml min^−1^. The eluate from the column was monitored, and 3D data were processed by SPD-M10A diode array detectors. All assigned peaks were identified by co-injection test with authentic samples and compared with UV spectral data.

### 2.2. Animals and Drug Treatments

Six-week-old male Wistar rats were purchased from Japan SLC Inc. (Hamamatsu, Japan) and kept in an automatically controlled room (temperature about 23°C and humidity about 60%) with a conventional dark/light cycle. At the age of 7 weeks, 5/6 nephrectomy was performed under anesthesia with sodium pentobarbital (50 mg kg^−1^ body weight, i.p.) by ablation of approximately 2/3 of the left kidney, and then removal of the right kidney by ligation of renal artery, vein and ureter 1 week later. As adaptive response to the renal ablation, remnant kidney undergo hypertrophy, resulting in glomerular hyperfiltration and hypertension, and proceed proteinuria, azotemia and renal fibrosis with glomerular sclerosis and tubulointerstitial scarring over time [10, 11]. After recovery from the operation (after 1 week), the animals were divided into three groups (a control and two treatment groups), avoiding any inter-group differences in blood urea nitrogen levels. A normal group of rats having undergone a sham operation was also included. During the experimental period, the normal (*n* = 5) and control (*n* = 15) groups were fed a standard chow, and the other two groups (*n* = 12, resp.) were fed a standard chow containing keishibukuryogan at a dose of 1% w/w or 3% w/w. The consumption of diet was kept at the same amount (30 g rat^−1^ day^−1^). After 4 weeks of treatment, the rats were sacrificed and blood samples were obtained. Tissues were quickly frozen and kept at −80°C until analysis.

All experimental procedures were performed in accordance with the standards established by the “Guide for the Care and Use of Laboratory Animals at the University of Toyama.”

### 2.3. Analysis of Serum and Urine Samples

Serum levels of creatinine and urea nitrogen were determined using commercial kits (CRE-EN Kainos and BUN Kainos purchased from Kainos Laboratories, Inc., Tokyo, Japan). Urinary protein content was determined using commercial kits (Micro TP-test, Wako Pure Chemical, Osaka, Japan). 

### 2.4. Reverse Transcriptase-Polymerase Chain Reaction (RT-PCR)

Total RNA was extracted using the RNeasy Mini Kit (QIAGEN K.K., Tokyo, Japan). cDNA was synthesized from 2 *μ*g of total RNA using an oligo dT primer (Invitrogen, Tokyo, Japan) and reverse transcriptase (SuperScript II, Invitrogen). PCR was performed using reverse transcription products (cDNA), each primer and a TAKARA Ex Taq PCR kit (Takara Shuzo Co. Ltd., Kyoto, Japan). The sequences of primers and PCR conditions are shown in Table 1. cDNA was amplified using a Thermal Cycler (Perkin-Elmer Cetus, Foster City, CA, USA). The number of cycles were predetermined for each pair of primers in order to avoid the PCR plateau phase. The parallel expression of glyceraldehyde-3-phosphate dehydrogenase (GAPDH) mRNA was tested as an internal standard. The PCR products were analyzed in 1.5% agarose gel along with a 100 bp DNA ladder, and the bands were scanned using a computer analysis system (H.P. Scan jet 4P and ATTO Densitograph ver. 4) and normalized to the corresponding GAPDH mRNA signal from the same sample.

### 2.5. Histological Study

For light microscopy, tissues were fixed with formalin (10%) and embedded in paraffin. Two *μ*m sections were stained with Azan stain, and the extent of tubulointerstitial changes was estimated.

### 2.6. Statistical Analysis

All values were presented as mean ± SE, and were analyzed by one-way analysis of variance (ANOVA) followed by Dunnett's test. *P *< .05 were accepted as statistically significant.

## 3. Results

### 3.1. Body and Kidney Weights

Changes in body and kidney weights are summarized in Table 2. The body weight of 5/6 nephrectomized rats was significantly lower than that of the normal rats. Body and kidney weights did not differ among the three nephrectomized groups, that is, control, 1% keishibukuryogan and 3% keishibukuryogan groups.

### 3.2. Renal Function

Table 3 shows the effects of keishibukuryogan on renal functional parameters. Compared with the normal group, the control group had significantly increased serum urea nitrogen and creatinine levels. Treatment with keishibukuryogan did not change the serum creatinine levels, but the 3% keishibukuryogan group showed a significant reduction in serum urea nitrogen compared with the control group. Similarly, the urinary protein content in the 3% keishibukuryogan group was reduced significantly.

### 3.3. RT-PCR

**Figure 2:**
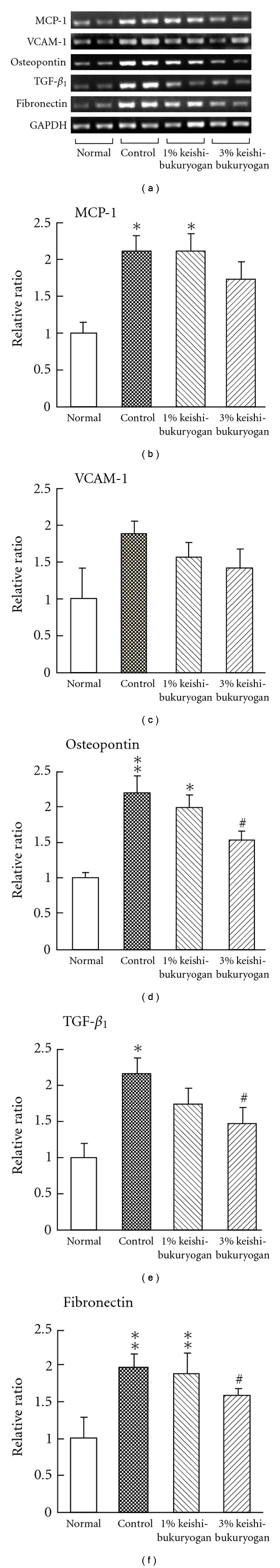
Effects of keishibukuryogan on mRNA levels in the remnant kidney of nephrectomized rats. (a) Typical example of each PCR product, (b) MCP-1, (c) VCAM-1, (d) osteopontin, (e) TGF-*β*_1_ and (f) fibronectin mRNA expression relative to normal rats. Statistical significance: *P < .05, **P < .01 versus normal rats, ^#^ *P *< .05 versus nephrectomized control rats.

Figure 2 shows the effects of keishibukuryogan on renal mRNA levels of monocyte chemoattractant protein-1 (MCP-1), vascular cell adhesion molecule-1 (VCAM-1), osteopontin, TGF-*β*_1_ and fibronectin. In nephrectomized control rats, significant increases in MCP-1, osteopontin, TGF-*β*_1_ and fibronectin expressions were observed compared with normal rats. The administration of 3% keishibukuryogan significantly suppressed osteopontin, TGF-*β*_1_ and fibronectin mRNA levels, while it showed a tendency to decrease MCP-1 and VCAM-1 mRNA levels without statistical significance.

### 3.4. Histological Examination

**Figure 3:**
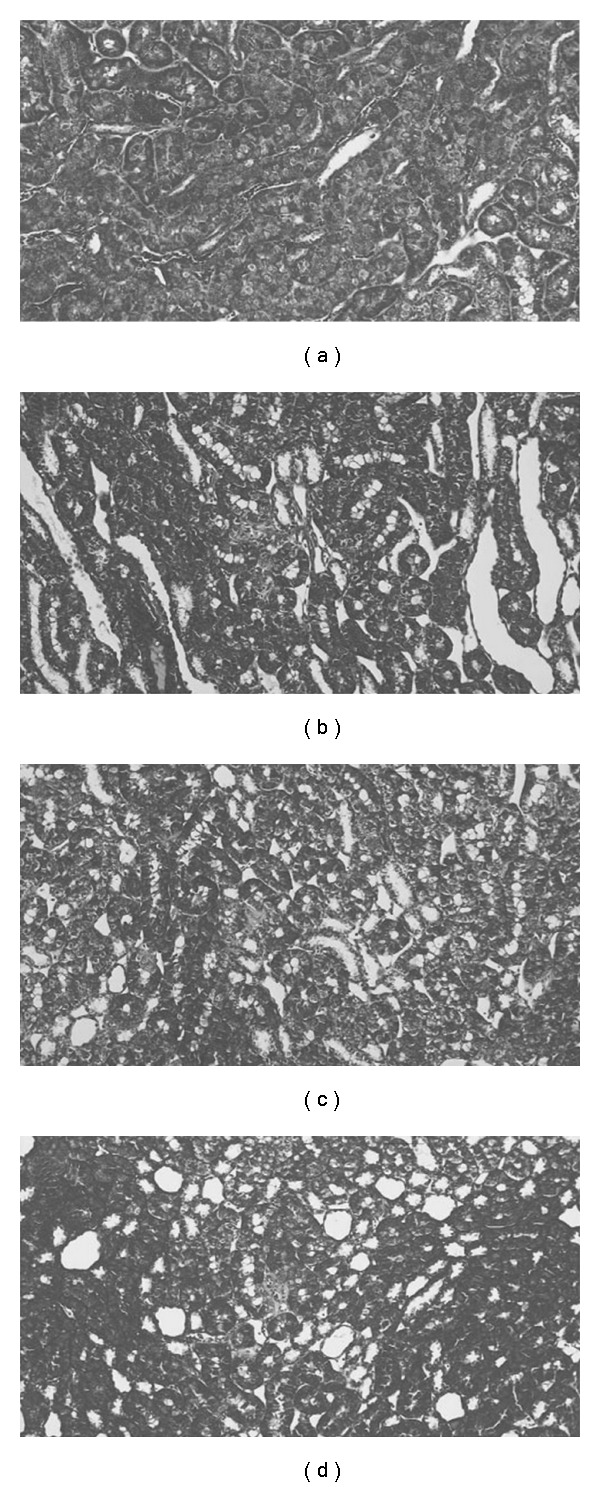
Representative photomicrographs of Azan-stained section in normal rats (a), nephrectomized rats in the control (b), 1% keishibukuryogan (c), 3% keishibukuryogan (d). Magnification 200X.

At 5 weeks after 5/6 nephrectomy, relatively mild tubulointerstitial fibrosis was observed (Figures 3(b)–3(d)). Such areas were virtually absent in the normal group (Figure 3(a)). Tubular dilation was observed in the 5/6 nephrectomized groups (Figures 3(b)–3(d)); there were no obvious differences among these groups.

## 4. Discussion

The 5/6 nephrectomy model, a well-characterized model of CRF, features proteinuria and renal dysfunction in association with glomerulosclerosis and tubulointerstitial fibrosis [12], and has been widely used to investigate the effects of therapeutic strategies and the pathogenic mechanisms of progressive renal failure.

Among the renal structural changes, tubulointerstitial fibrosis is known to be a common histological appearance in patients with CRF, and it is thought to be correlated with the decline of renal function and prognosis [13, 14]. It was reported that tubulointerstitial fibrosis is severe at 12 weeks after 5/6 nephrectomy, with large areas displaying tubular dilation, intratubular cast formation and tubular atrophy, and that it was relatively mild at 3 and 6 weeks after 5/6 nephrectomy [15]. Similar results were observed in the present study, with mild tubulointerstitial fibrosis at 5 weeks after 5/6 nephrectomy being the only change compared with normal rats, and keishibukuryogan treatment also showed similar features. Additionally, glomerular sclerosis and arteriolar hyalinosis were inconspicuous in the three nephrectomy groups. So, it indicates that severe structural changes did not develop at this point (Figure 3).

The pathogenesis of the development of CRF includes macrophage infiltration [16]. Macrophages are known to participate in inflammatory reaction, producing a variety of cytokines and growth factors [17]. Many studies have reported that the number of macrophages as revealed by immunostaining for ED1 increased remarkably in the remnant kidney of rats subjected to 5/6 nephrectomy [16, 18–20], and it was also observed that macrophage infiltration and proliferation occurs early (2–4 weeks after surgery), preceding the development of glomerulosclerosis and tubulointerstitial fibrosis [16, 18, 21]. On the other hand, the reduction of inflammatory cell infiltration with some agents was reported to be associated with the prevention of progressive renal failure [22, 23]. Therefore, it is considered that macrophage infiltration is one of the most important events, preceding the development of severe renal injury, and that the suppression of macrophage infiltration would be a useful therapeutic approach.

The infiltration of macrophages is induced by the upregulation of cell adhesion molecules and chemokines. From recent studies using several animal models such as glomerulonephritis, unilateral ureteral obstruction, diabetic nephropathy and 5/6 nephrectomy [22, 24–27], the expressions of MCP-1, VCAM-1 and osteopontin have been shown to be elevated. Additionally, increases of these gene expressions have been suggested to contribute to renal damage by promoting macrophage infiltration. Therefore, in this study, we focused on the early stage of progressive renal failure before the development of severe renal injury, and examined whether keishibukuryogan could suppress the gene expressions associated with macrophage recruitment in the remnant kidney of rats at 5 weeks after 5/6 nephrectomy. Our results showed that the administration of 3% keishibukuryogan significantly suppressed osteopontin mRNA levels. Osteopontin, a potent chemotactic and adhesion molecule for monocyte/macrophage, has been recently shown to have strong association with focal macrophage infiltration in a number of experimental models of renal injury, suggesting a pathologic role in progressive renal injury [28–30]. Keishibukuryogan also induced a decreasing tendency in both MCP-1 and VCAM-1 mRNA expressions, although not statistically significant. These results suggest that keishibukuryogan treatment inhibits macrophage infiltration by suppression of mRNA levels related to macrophage recruitment, although further studies are needed to clarify whether keishibukuryogan suppress the macrophage infiltration by immunostaining for ED1.

Accumulation of extracellular matrix (ECM) proteins, a conspicuous finding accompanying the progression of renal failure, is also included in the pathogenesis of progressive renal failure [31, 32]. Kliem et al. [19] reported that progressive interstitial accumulation of ECM proteins such as fibronectin, type IV collagen and laminin occurred, in parallel with monocyte/macrophage influx, in the 5/6 nephrectomized model. Macrophages may participate in the development of fibrosis by the release of fibrogenic cytokines such as TGF-*β*_1_ and interleukin-1 [33]. TGF-*β*_1_ has been implicated as playing a central role in the regulation of the overdeposition of ECM proteins [32, 34]. Immunohistochemical examination TGF-*β*_1_ and fibronectin expressions has shown their increase predominantly along fibrous tissue in the remnant kidney of 5/6 nephrectomized rats [35]. Furthermore, increased TGF-*β*_1_ mRNA expression has been observed at 4 weeks, and was sustained at 12 weeks after 5/6 nephrectomy [25]. From these findings, suppression of TGF-*β*_1_ and ECM protein overdeposition is also thought to be a useful therapeutic approach. Previously, we demonstrated that keishibukuryogan suppressed TGF-*β*_1_ and fibronectin protein expressions in the kidney of diabetic rats [9]. Therefore, we examined their gene expressions in the remnant kidney of 5/6 nephrectomized rats. Our results showed that keishibukuryogan treatment significantly suppressed mRNA levels of TGF-*β*_1_ and fibronectin, suggesting that keishibukuryogan exerts beneficial effects on the kidney by inhibiting ECM protein accumulation induced by TGF-*β*_1_, independently of the primary disease.

Oxidative stress is widely recognized to involve the pathogenesis of CRF. As one of causal link to urinary protein excretion and renal dysfunction, impairment of glomerular basal membrane due to lipid peroxidation induced by enhanced oxidative stress has been suggested [36]. Previously, we reported that keishibukuryogan decreased lipid peroxidation and elevated superoxide dismutase activity in the kidney [9, 37]. On the other hand, keishibukuryogan has been demonstrated to improve blood circulation and vasoendothelial disorders, and discussed the link to anti-oxidative effects of keishibukuryogan [7, 8, 38, 39], from the viewpoint in which keishibukuryogan has been traditionally used for improvement of blood stagnation syndrome. Therefore, we speculate that keishibukuryogan improve oxidative conditions and microcirculation in the kidney of CRF, and these effects may contribute, at least in parts, to the attenuation of serum urea nitrogen and urinary protein excretion.

The number of patients suffering from CRF is increasing year by year. Interventions using angiotensin-converting enzyme (ACE) inhibitors, angiotensin receptor blockers and protein restriction, aimed at slowing the progression of CRF, are widely accepted. On the other hand, it has also been reported that the use of ACE inhibitors induced hyperkalemia, a life-threatening complication [40]. Keishibukuryogan has long been prescribed for patients, and serious adverse reactions have not been reported. Additionally, in the present study, body weight and survival rate were not adversely affected by the administration of keishibukuryogan. As a result, keishibukuryogan has widened expectations for its clinical application, although further clinical trials on possible adverse reactions will still be needed to ensure its safe use for patients with renal dysfunction.

**Figure 4:**
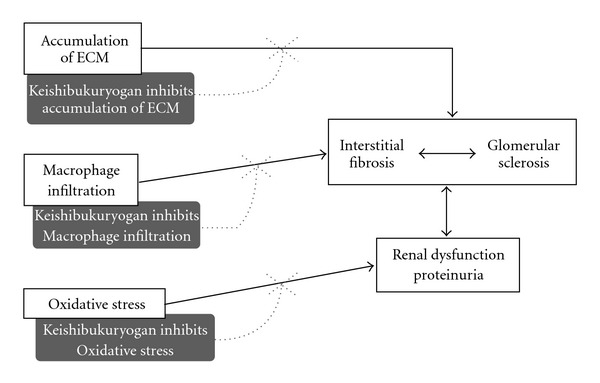
Hypothetical representation of the effects of keishibukuryogan on renal failure.

In summary, our present results provided evidence that keishibukuryogan suppressed gene expressions associated with macrophage infiltration and renal fibrosis in the remnant kidney of rats subjected to 5/6 nephrectomy, preceding the development of severe renal failure. Moreover, keishibukuryogan improved proteinuria and renal dysfunction. We summarized our hypothetical representation that explain the effects of keishibukuryogan on renal failure (Figure 4). Although further experiments are called for to definitively determine whether keishibukuryogan can ameliorate the severe functional and structural changes that take place longitudinally after 5/6 nephrectomy, the results of this study suggested that keishibukuryogan treatment at the early stage of progressive renal failure is useful for slowing down CRF progression.

## Figures and Tables

**Table 1:** Summary of primer sequences, PCR conditions and size of amplified fragments.

Genes	Orientation	Sequences (5′ to 3′)	Annealing	Size
MCP-1	Sense	GCC AGA TCT CTC TTC CTC CA	62°C, 60 s	432 bp
	Antisense	GAG GTG GTT GTG GAA AAG AG		
VCAM-1	Sense	CTG ACC TGC TCA AGT GAT GG	60°C, 50 s	260 bp
	Antisense	GTG TCT CCC TCT TTG ACG CT		
Osteopontin	Sense	AGA GGA GAA GGC GCA TTA CA	61°C, 60 s	498 bp
	Antisense	GCA ACT GGG ATG ACC TTG AT		
TGF-*β*_1_	Sense	TAC AGG GCT TTC GCT TCA GT	61°C, 60 s	394 bp
	Antisense	TGG TTG TAG AGG GCA AGG AC		
Fibronectin	Sense	TTA TGA CGA CGG GAA GAC CTA	56°C, 60 s	295 bp
	Antisense	GGC TGG ATG GAA AGA TTA CTC		
GAPDH	Sense	GTG AGG TGA CCG CAT CTT CT	56°C, 60 s	278 bp
	Antisense	TGG AAG ATG GTG ATG GGT TT		

MCP-1, monocyte chemoattractant protein-1; VCAM-1, vascular cell adhesion molecule-1; TGF-*β*_1_, transforming growth factor-*β*_1_; GAPDH, glyceraldehyde-3-phosphate dehydrogenase.

**Table 2:** Effects of keishibukuryogan on body and tissue weights.

Group	Body weight (g)	Kidney weight (g/100 g BW)
Normal rats	349 ± 14	
Nephrectomized rats		
Control	314 ± 22*	0.381 ± 0.062
1% keishibukuryogan	317 ± 17*	0.348 ± 0.040
3% keishibukuryogan	319 ± 21*	0.371 ± 0.049

Statistical significance: *P < .05 versus normal rats.

**Table 3:** Effects of keishibukuryogan on biochemical parameters.

Group	Serum urea nitrogen (mg dl^−1^)	Serum creatinine (mg dl^−1^)	Urinary protein excretion (mg mg^−1^ Cr)
Normal rats	16.9 ± 2.7	0.485 ± 0.152	1.94 ± 0.21
Nephrectomized rats			
Control	35.6 ± 4.5*	0.770 ± 0.083*	2.79 ± 0.52
1% keishibukuryogan	32.2 ± 4.1*	0.769 ± 0.068*	2.35 ± 0.60
3% keishibukuryogan	29.3 ± 4.9^∗,###^	0.738 ± 0.081*	1.32 ± 0.36^#^

Statistical significance: *P < .01 versus normal rats, ^#^ *P *< .05, ^##^ *P *<  .01 versus nephrectomized control rats.
